# Infiltration of TIM4-positive intratumoral macrophages serves as an adverse prognostic factor in breast cancer

**DOI:** 10.1007/s12282-026-01825-8

**Published:** 2026-01-21

**Authors:** Mio Yamaguchi-Tanaka, Kiyoshi Takagi, Miyu Takahashi, Ai Sato, Yuto Yamazaki, Minoru Miyashita, Takashi Suzuki

**Affiliations:** 1 Department of Pathology and Histotechnology, Tohoku University Graduate School of Medicine, 2-1 Seiryo-machi, Aoba-ku, Sendai, Miyagi 980-8575 Japan; 2 Department of Pathology, Tohoku University Hospital, Sendai, Miyagi 980-8575 Japan; 3 Breast and Endocrine Surgical Oncology, Tohoku University Graduate School of Medicine, Sendai, Miyagi 980-8575 Japan; 4 Anatomic Pathology, Tohoku University Graduate School of Medicine, Sendai, Miyagi 980-8575 Japan

**Keywords:** Breast cancer, Macrophage, TIM4, Immunohistochemistry, Prognostic factor

## Abstract

**Background:**

T-cell immunoglobulin and mucin domain containing protein 4 (TIM4), a phosphatidylserine receptor primarily expressed on antigen-presenting cells, has been implicated in phagocytosis and immune regulation in various diseases, including malignancies. However, the significance of TIM4 in the breast cancer microenvironment remains unclear. In this study, we investigated the localization and clinical significance of TIM4 in breast cancer.

**Methods:**

We immunolocalized TIM4 in human breast carcinoma tissues using immunohistochemistry (IHC) and multiplex fluorescence-immunohistochemistry (F-IHC) and examined its correlation with clinicopathological parameters and clinical outcomes.

**Results:**

TIM4 was highly expressed in both carcinoma cells and stromal cells in human breast carcinoma tissues. Multiplex F-IHC revealed that TIM4 was co-localized with CD68, a macrophage marker, whereas no co-localization was observed between TIM4 and CD80 (an M1 macrophage marker) or CD163 (an M2 macrophage marker). Prognostic analysis of 171 breast carcinoma tissues by IHC revealed that infiltration of TIM4-positive stromal cells was associated with an aggressive tumor phenotype, including increased proliferative and invasive potential, as well as poorer clinical outcomes. In contrast, TIM4 immunoreactivity in carcinoma cells showed no significant correlation with clinical outcomes.

**Conclusions:**

These findings suggest that infiltration of TIM4-positive macrophages serves as a strong prognostic indicator in breast cancer and that TIM4 may represent a novel marker for tumor-promoting macrophages.

**Supplementary Information:**

The online version contains supplementary material available at 10.1007/s12282-026-01825-8.

## Introduction

Breast cancer is one of the most common cancers in women worldwide. In 2022, breast cancer had the highest age-standardized incidence rate and was the second leading cause of cancer-related death, following lung cancer [1]. To date, extensive research has focused on identifying new therapeutic targets and biomarkers in breast cancer cells. However, the breast cancer microenvironment consists not only of cancer cells but also of various components, including stromal cells, with interactions between tumor cells and these elements playing a critical role in tumor progression [2, 3]. In particular, tumor-associated macrophages (TAMs) are a major component of the tumor microenvironment and contribute to tumor malignancy by regulating cancer cell behavior, immune responses, extracellular matrix remodeling, and angiogenesis.

T-cell immunoglobulin and mucin domain containing protein 4 (TIM4) is a type I transmembrane protein that functions as a receptor for phosphatidylserine [4]. It is primarily expressed on antigen-presenting cells (APCs), including macrophages and dendritic cells, and is involved in the recognition and efferocytosis of apoptotic cells [5]. TIM4 also serves as a ligand for TIM1 and acts as a co-stimulatory molecule that regulates T-cell expansion and survival [6]. Furthermore, TIM4 has also been found to be expressed in lymphocytes, such as natural killer T cells and B cells, in addition to APCs, and the function and significance of TIM4 expression on immune cells in regulating immune responses across various diseases and tissues have been increasingly recognized [7, 8]. Importantly, previous studies suggest that TIM4 is involved in tumor progression. TIM4 expression in APCs has been reported to contribute to an immunosuppressive microenvironment, thereby promoting peritoneal metastasis of ovarian cancer, as well as metastasis to serous body cavities and chemoresistance in melanoma, lung, and colon cancers [9–11]. Baghdadi et al. demonstrated that antibody-mediated inhibition of TIM4 enhanced the therapeutic efficacy of cancer vaccines against melanoma in an in vivo model [12]. In contrast, TIM4-positive B cells have been shown to reduce melanoma metastasis and tumor growth in an interferon γ-dependent manner in vivo [8]. However, the clinicopathological significance of TIM4 expression in stromal cells within various tumor microenvironments, including breast cancer, has not yet been elucidated.

Furthermore, recent studies have confirmed that TIM4 is also expressed in various malignant tumor cells and is closely associated with tumor progression, particularly in lung cancer [13–16]. Although TIM4 has been detected in human breast cancer cells, its clinical significance and biological role in breast cancer remain unclear [13]. Therefore, we investigated the immunolocalization of TIM4 in human breast carcinoma tissues using immunohistochemistry, and analyzed its immunoreactivity in relation to clinicopathological parameters and clinical outcomes.

## Materials and methods

### Patients and tissues

A total of 171 invasive breast cancer specimens were collected from patients who underwent surgical resection at Tohoku University Hospital between 2006 and 2008. All tissues were fixed in 10% neutral-buffered formalin and embedded in paraffin. The clinicopathological characteristics of the cases are summarized in Table 1. Histological grade data were missing for two of the 171 cases, resulting in 169 cases available for histological grade analysis. Clinical outcomes were assessed based on disease-free survival (defined as the time from surgery to locoregional or distant metastasis) and breast cancer-specific survival, with median follow-up periods of 63 months (range: 3–108 months) and 64 months (range: 3–108 months), respectively. This study was approved by the Ethics Committee of Tohoku University School of Medicine, and was conducted in accordance with the institutional ethical guidelines.

**Table 1:** Clinicopathological characteristics of the cases used in this study (n = 171)

		n
Age^a^		55 (27–87)
Menopausal status	Pre-	66
	Post-	105
pT	pT1	111
	pT2-4	60
Lymph node metastasis	Negative	117
	Positive	54
Stage	1	95
	2	46
	3	30
Histological grade	1	61
	2	76
	3	32
ER	Negative	30
	Positive	141
PR	Negative	56
	Positive	115
HER2	Negative	144
	Positive	27
Ki67 LI^a^		12 (1–72)

^a^Data was presented as median (minimum–max). All other values represent the number of cases

*HER2* human epidermal growth factor receptor type 2, *LI* labeling index, *pT* pathological T factor

### Immunohistochemistry

Information about the primary antibodies is listed in Table 2. Antigen retrieval for TIM4 was carried out using an autoclave at 121 °C for 10 min in Tris/EDTA buffer (pH 9). Immunoreactivity was visualized using the Histofine kit (Nichirei Biosciences, Tokyo, Japan) with 3,3′-diaminobenzidine (DAB) as the chromogen, followed by hematoxylin counterstaining [17]. The immunohistochemical evaluation of estrogen receptor (ER), progesterone receptor (PR), human epidermal growth factor receptor 2 (HER2), and Ki67 labeling index (LI) was performed according to previously published methods [18].

**Table 2:** Information on the antibody used in this study

Target	Antibody^a^	Company	Dilution
TIM4	Goat polyclonal (AF2929)	R&D	1:100
CD68	Mouse monoclonal (PG-M1)	DAKO	1:200
CD11c	Mouse monoclonal (2F1C10)	Proteintech	1:10,000
CD20	Mouse monoclonal (L26)	DAKO	Ready to use
CD80	Mouse monoclonal (37,711)	R&D	1:100
CD163	Mouse monoclonal (10D6)	Leica	1:100

^a^Clone number or catalog number were given for monoclonal antibody and polyclonal antibody, respectively

*TIM4* T-cell immunoglobulin and mucin domain containing protein 4

### Scoring of immunoreactivities

The extent of TIM4-positive stromal cell infiltration (TIM4-stroma) was classified into three levels: 0 (no detectable foci), 1 (a few small foci), and 2 (multiple large foci) according to a previous study [19, 20], and was subsequently dichotomized as low (scores 0–1) or high infiltration (score 2). TIM4 expression in carcinoma cells (TIM4-carcinoma) was considered positive when more than 10% of tumor cells exhibited immunoreactivity, based on the criteria previously used to evaluate immunostaining [21–23]. Immunostaining was evaluated independently by two authors in a blinded manner. In cases of disagreement, the slides were re-reviewed to reach a final consensus.

### Fluorescence-immunohistochemistry (F-IHC)

Specimens with high infiltration of TIM4-positive cells, along with positive control tissues, were selected for F-IHC analysis. Information about the primary antibodies is listed in Table 2. Antigen retrieval was performed using an autoclave at 121 °C for 10 min in Tris/EDTA buffer (pH 9). Tissue sections were mounted with DAPI-containing mounting medium (Vector Laboratories, Newark, CA, USA). Fluorescence signals were visualized using the BZ-X810 all-in-one fluorescence microscope (Keyence Corporation, Osaka, Japan).

For double F-IHC, tissues were incubated overnight with primary antibodies against TIM4 and either CD68, CD11c, or CD20. Following this, the samples were incubated with Donkey Anti-Goat IgG H&L (Alexa Fluor® 488, 1:200, cat. no. ab150129, Abcam, Cambridge, UK), and then with Alexa Fluor 594-AffiniPure anti-mouse IgG (1:100, cat. no. 115–585-003, Jackson ImmunoResearch, West Grove, PA, USA). Multiplex F-IHC was performed using the Opal 4-Plex Manual Detection Kit (Akoya Biosciences, Marlborough, MA, USA). Briefly, tissues were incubated overnight with primary antibodies, after which the antigen–antibody complexes were labeled with horseradish peroxidase (HRP) using the Histofine Kit (Nichirei), followed by incubation with tyramide-conjugated fluorophores (Opal 520, Opal 570, and Opal 690). The tyramide-fluorophore is activated by reaction with HRP and forms covalent bonds with tyrosine residues in the vicinity of the target protein. Antibodies were removed by heating steps using autoclave (110 °C, 5 min, citrate buffer, pH 6), leaving only the fluorophores bound to the tissue. This process was repeated for each target, using a different Opal fluorophore each time.

### Statistical analysis

Statistical analyses were conducted using JMP Student Edition 18.2.0 (SAS Institute, Cary, NC, USA). Associations between TIM4 immunoreactivity and clinicopathological parameters were evaluated using the chi-square (χ^2^) test or the Wilcoxon rank sum test. Survival curves were generated using the Kaplan–Meier method and compared with the log-rank test. A p-value of less than 0.05 was considered statistically significant.

## Results

### Immunolocalization of TIM4 in human breast carcinoma tissues

**Fig. 1:**
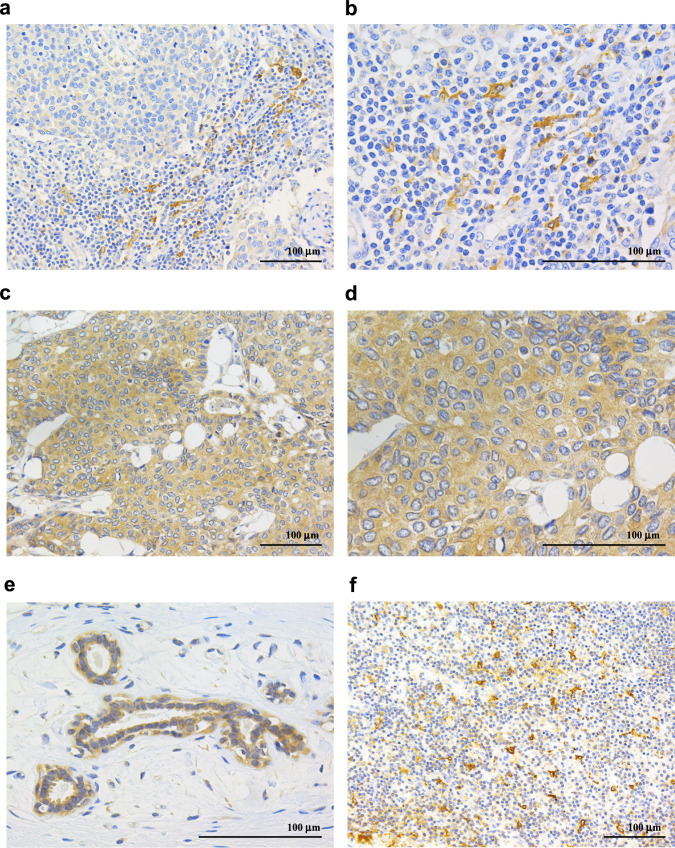
Immunohistochemistry for TIM4 in human breast carcinoma tissues. TIM4 immunoreactivity was observed in the membranes and cytoplasm of stromal cells (**a**; × 200, **b**; × 400) and carcinoma cells (**c**; × 200, **d**; × 400), and was also detected in the normal breast epithelium (**e**; × 400). Human lymph node tissue was used as a positive control for TIM4 staining (**f**; × 200). Scale bar = 100 μm

TIM4 immunoreactivity was detected in the membranes and cytoplasm of stromal cells (Fig. 1a, b) as well as in breast carcinoma cells (Fig. 1c, d). Notably, TIM4-positive stromal cells were frequently observed in areas with dense lymphocyte infiltration. TIM4 expression was also present in the normal breast epithelium (Fig. 1e). A lymph node served as a positive control for TIM4 immunostaining (Fig. 1f).

**Fig. 2:**
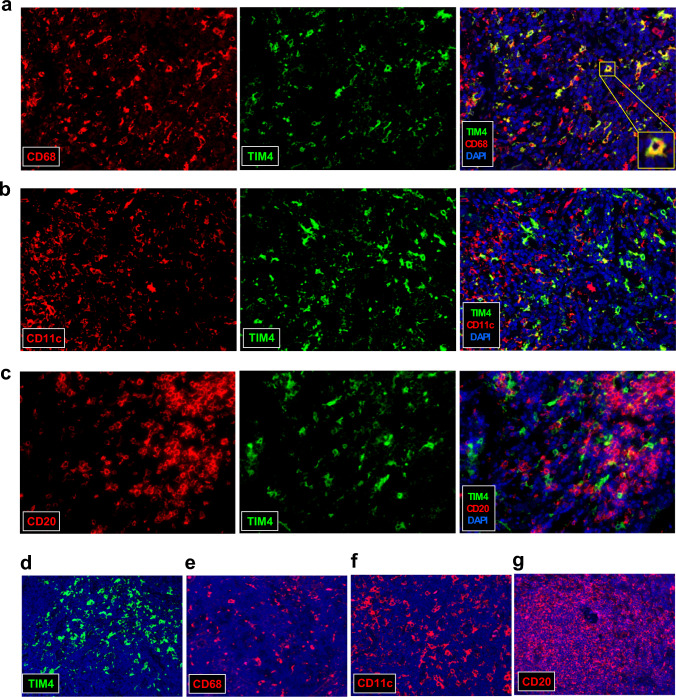
Double F-IHC for TIM4 and surface markers of immune cells in breast carcinoma tissues. Double F-IHC staining was performed for TIM4 (green) and CD68 (**a**, red), CD11c (**b**, red), and CD20 (**c**, red), with DAPI (blue) used for nuclear counterstaining, in human breast carcinoma tissues. Human lymph node tissue was used as a positive control for TIM4 (**d**), CD68 (**e**), CD11c (**f**), and CD20 (**g**) staining. All images were acquired at 400 × magnification

Next, double F-IHC was performed on breast carcinoma tissues to identify the types of stromal cells expressing TIM4. Previous studies have reported that TIM4 is primarily expressed in macrophages, dendritic cells, and B cells, and is involved in tumor progression [8–12]. In this study, we examined the co-localization of TIM4 with CD68 (a macrophage marker), CD11c (a dendritic cell marker), and CD20 (a B cell marker). TIM4 showed strong colocalization with CD68 (Fig. 2a), whereas only limited colocalization was observed with CD11c (Fig. 2b) and CD20 (Fig. 2c), suggesting that TIM4 is predominantly expressed in macrophages within the breast cancer microenvironment. Lymph nodes were used as positive controls for TIM4 (Fig. 2d), CD68 (Fig. 2e), CD11c (Fig. 2f), and CD20 (Fig. 2g) immunofluorescence. TIM4 was partially co-localized with CD68, CD11c, and CD20 in lymph node tissues (data not shown).

**Fig. 3:**
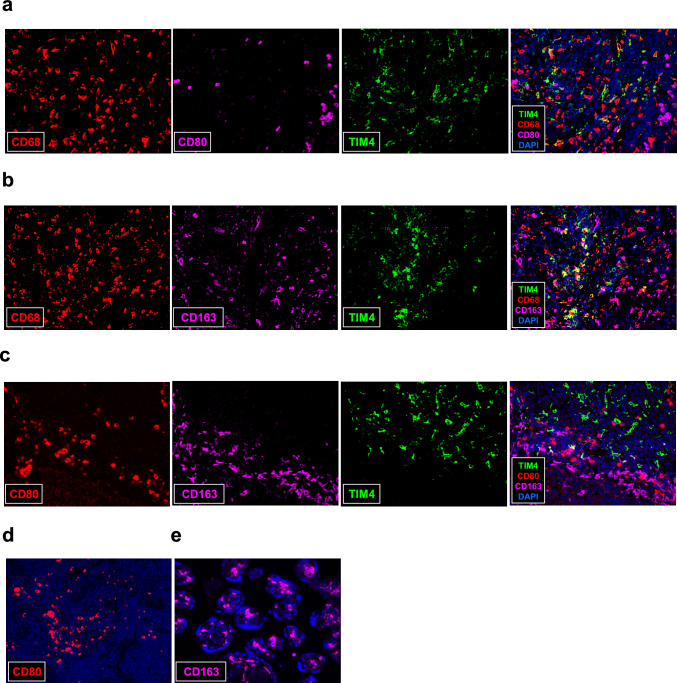
Multiplex F-IHC for TIM4 and macrophage markers in breast carcinoma tissues. **a**, **b** Multiplex F-IHC staining was performed for TIM4 (green), CD68 (red), and CD80 (**a**, purple) or CD163 (**b**, purple) in human breast carcinoma tissues. **c**: Multiplex F-IHC staining was performed for TIM4 (green), CD80 (red), and CD163 (purple) in human breast carcinoma tissues. **d**, **e** Human lymph node (**d**) and placenta tissues (**e**) were used as positive controls for CD80 and CD163, respectively. DAPI (blue) was used for nuclear counterstaining. All images were acquired at 400 × magnification

Since TIM4 expression was observed in intratumoral macrophages, we further investigated the macrophage subtypes expressing TIM4. Macrophages are generally classified into M1 or M2 phenotypes based on their function. Multiplex F-IHC for TIM4, CD68, and CD80 (an M1 macrophage marker, Fig. 3a), or CD163 (an M2 macrophage marker, Fig. 3b), revealed that expression of both CD80 and CD163 was negligible in TIM4⁺ CD68⁺ cells. Similarly, multiplex F-IHC for TIM4, CD80, and CD163 (Fig. 3c) showed that TIM4, CD163, and CD80 were localized in distinct cell populations. Lymph node and placenta tissues were used as positive controls for CD80 (Fig. 3d) and CD163 (Fig. 3e) immunostaining, respectively.

### Correlation between TIM4 immunoreactivity and clinicopathological parameters

In the present study, 13% (22 out of 171 cases) and 44% (76 out of 171 cases) were categorized as having high infiltration of TIM4-positive stromal cells (TIM4-stroma) and as positive for TIM4 in carcinoma cells (TIM4-carcinoma), respectively. The associations between clinicopathological parameters and immunoreactivity of TIM4 are summarized in Table 3. Infiltration of TIM4-positive stromal cells was significantly correlated with lymph node metastasis (P = 0.046), histological grade (P = 0.0007), and Ki67 LI (P < 0.0001), while it was inversely correlated with ER (P = 0.0002) and PR (P = 0.0001). No significant correlations were found between TIM4-carcinoma and clinicopathological factors. No correlation was observed between infiltration of TIM4-positive stromal cells and TIM4-carcinoma expression (P = 0.92).

**Table 3:** Clinicopathological characteristics of TIM4 in breast carcinoma tissues (n = 171)

	Infiltration of TIM4 positive stroma cells (TIM4-stroma)			TIM4-carcinoma		
	Low	High	P	Negative	Positive	P
	(n = 149)	(n = 22)		(n = 95)	(n = 76)	
Age^a^	55 (27–87)	58 (33–82)	0.53	56 (27–87)	55 (29–85)	0.32
Menopausal status						
Pre-	59	7	0.48	37	29	0.92
Post-	90	15		58	47	
pT						
pT1	100	11	0.12	61	50	0.83
pT2–4	49	11		34	26	
Lymph node metastasis						
Negative	106	11	**0.046**	68	49	0.32
Positive	43	11		27	27	
Stage						
1	87	8	0.14	53	42	0.98
2	37	9		25	21	
3	25	5		17	13	
Histological grade						
1	59	2		40	21	
2	66	10	**0.0007**	37	39	0.14
3	22	10		18	14	
Estrogen receptor						
Negative	20	10	**0.0002**	15	15	0.50
Positive	129	12		80	61	
Progesterone receptor						
Negative	41	15	**0.0001**	30	26	0.72
Positive	108	7		65	50	
HER2						
Negative	128	16	0.11	83	61	0.21
Positive	21	6		12	15	
Ki67 LI^a^	10 (1–72)	22 (6–49)	** < 0.0001**	10 (1–72)	12.5 (1–53)	0.57
TIM4-carcinoma						
Negative	83	12	0.92	–	–	–
Positive	66	10		–	–	

^a^Data was presented as median (minimum–max). All other values represent the number of cases. P < 0.05 was considered significant and described as bold

*HER2* human epidermal growth factor receptor type 2, *LI* labeling index, *pT* pathological T factor, *TIM4* T-cell immunoglobulin and mucin domain containing protein 4

### Association between clinical outcome and immunoreactivity of TIM4 in breast carcinoma tissues

**Fig. 4:**
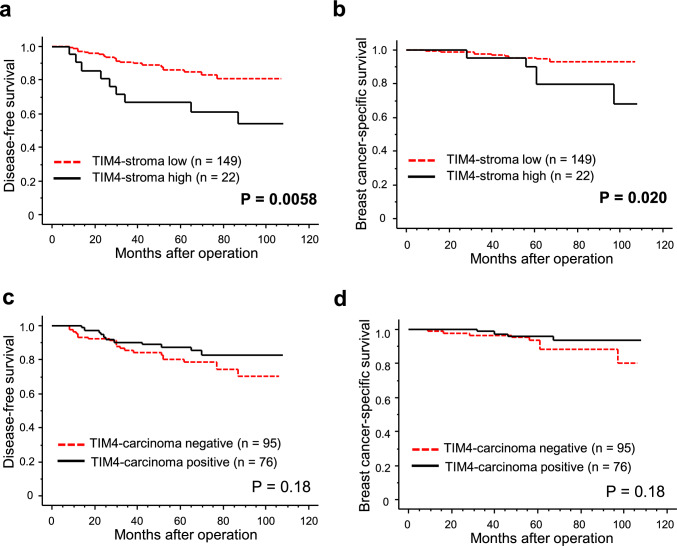
Prognostic analysis according to TIM4 immunoreactivity in stromal cells and carcinoma cells in human breast carcinoma tissues. Disease-free (**a, c**) and breast cancer-specific survival (**b, d**) based on the infiltration of TIM4-positive stromal cells (**a, b**; TIM4-stroma) and TIM4 immunoreactivity in carcinoma cells (**c, d**; TIM4-carcinoma) in 171 breast cancer patients. Survival curves were generated using the Kaplan–Meier method

Infiltration of TIM4-positive stromal cells was significantly associated with shorter disease-free survival (Fig. 4a, P = 0.0058) and breast cancer-specific survival (Fig. 4b, P = 0.020). In contrast, TIM4-carcinoma immunostaining showed no significant correlation with disease-free survival (Fig. 4c, P = 0.18) or breast cancer-specific survival (Fig. 4d, P = 0.18). In addition, we analyzed the association between TIMD4 mRNA expression, which encodes TIM4, and overall survival in patients with breast cancer using publicly available datasets. While analysis using the Kaplan–Meier Plotter (KM-Plotter) showed that high TIMD4 mRNA expression was associated with poor prognosis in breast cancer patients (P = 0.0015, Supplementary Fig. a), analyses using the GDC TCGA-BRCA database (P = 0.72, Supplementary Fig. b) and the Gene Expression Profiling Interactive Analysis 2 (GEPIA2) database (P = 0.69, Supplementary Fig. c) revealed no correlation between TIMD4 mRNA expression and patient prognosis.

The results of univariate and multivariate analyses are summarized in the Supplementary Table. Univariate analysis identified TIM4-stroma, pT, lymph node metastasis, histological grade, ER, PR, and Ki67 LI as significant prognostic factors for disease-free survival, but multivariate analysis showed that only pT (P = 0.0015) was independent. Similarly, for breast cancer–specific survival, TIM4-stroma, pT, lymph node metastasis, histological grade, PR, and Ki67 LI were significant in univariate analysis, whereas only pT (P = 0.018) remained independent.

## Discussion

In the present study, we first demonstrated the clinicopathological significance of TIM4 in human breast carcinoma tissues using immunohistochemistry and immunofluorescence. We found that TIM4 is highly expressed in both carcinoma cells and stromal cells, especially macrophages, in human breast carcinoma tissue. TIM4 has been reported to be expressed in APCs, including macrophages and dendritic cells, in various tissues such as tonsils, thymus, spleen, and lymph nodes [6, 24–26]. Previous studies on human malignancies have reported that macrophages in human serous body cavities express TIM4, and that various tumor cells—including those of breast, lung, clear cell renal cell carcinoma (CCRCC), pancreatic ductal adenocarcinoma (PDAC), and glioma—also express TIM4, as shown by immunohistochemistry [9, 13, 14, 27–30]. These findings reflect the diverse functions of TIM4 in influencing tumor cell behavior and the tumor microenvironment across different cancers. Although TIM4 is frequently reported to be expressed in DCs, its expression in DCs within breast carcinoma tissues was low. Notably, Caronni et al. reported that TIM4 is highly expressed in normal lung-resident DCs, but is downregulated in advanced tumors [31]. These observations suggest that TIM4 expression in DCs may be suppressed across various tumor microenvironments.

Infiltration of TIM4-positive macrophages was significantly associated with worse clinical outcomes, including shorter disease-free and breast cancer-specific survival.　To date, no studies have investigated the prognostic significance of TIM4-positive macrophages in human malignant tissues using immunohistochemistry. This study is the first to demonstrate that the immunohistochemical status of TIM4-positive macrophage infiltration serves as a strong prognostic indicator in breast cancer. Analysis using KM Plotter showed that high TIMD4 mRNA expression in breast carcinoma tissues was associated with poor prognosis in breast cancer patients, supporting part of the findings of this study. On the other hand, analyses using the GDC TCGA-BRCA database and the GEPIA2 database revealed no correlation between TIMD4 mRNA expression and patient prognosis. These discrepancies may be attributable to differences in the cohort characteristics used in each database. Furthermore, although public databases assess mRNA expression levels in whole breast carcinoma tissues, the present study evaluated protein expression in carcinoma cells and stromal cells using immunohistochemistry. Therefore, the differing results are not necessarily contradictory. Notably, this study demonstrated that infiltration of TIM4-positive stromal cells was a poor prognostic factor in breast cancer, whereas TIM4 expression in carcinoma cells was not associated with prognosis. Thus, although TIM4 has the potential to serve as a biomarker, its cellular localization should be carefully considered.

Interestingly, our multiplex F-IHC analysis showed that TIM4-positive macrophages exhibited negligible expression of both CD80 and CD163. While M1 macrophages typically express CD80 and CD86 and exert tumor-suppressive and pro-inflammatory functions, M2 macrophages—characterized by CD163 expression—are the predominant TAM phenotype in solid tumors and contribute to tumor progression [32]. A previous study demonstrated that TIM4 is highly expressed in M2-like tissue-resident macrophages [33]. Mechanistically, TIM4 promotes the differentiation of adipose tissue macrophages into M2 macrophages by suppressing the NF-κB signaling pathway [34]. Liang et al. further showed that PS generated by apoptotic cells induces M2 macrophage polarization through TIM4 activation both in vivo and in vitro [35]. On the other hand, in the present study, no co-localization of TIM4 with CD163, an M2 marker, was observed in breast carcinoma tissues, although TIM4-positive macrophages were a strong predictor of poor prognosis in breast cancer patients. Notably, recent technological advances have further revealed the complexity of macrophage heterogeneity within the tumor microenvironment, beyond the conventional M1/M2 classification [36, 37]. A novel subtype of TIM4-positive macrophages has been suggested, and these cells may serve as a new prognostic marker in breast cancer. In the future, their functional roles in breast cancer will likely be clarified through detailed analyses of soluble factor and surface marker expression using approaches such as single-cell analysis.

Infiltration of TIM4-positive macrophages was significantly correlated with lymph node metastasis, histological grade, and Ki67 LI, a marker of cell proliferation, while it was inversely correlated with ER and PR status. These findings indicate that TIM4-positive macrophages are highly infiltrated in breast cancers with an aggressive phenotype characterized by active proliferative and invasive potential. TIM4 is a receptor for PS and mediates the phagocytosis of apoptotic cells by binding to PS exposed on their surface [4]. TIM4-positive macrophages, have been reported to suppress antitumor immune activity [9–12]. TIM4 on APCs translocates to LAMP1⁺ phagosomes after engulfing apoptotic cells, where it interacts with AMPKα1, promoting excessive degradation of tumor antigens and suppressing cross-priming of cytotoxic T cells, thereby weakening chemotherapy-induced antitumor immunity [9]. Chow et al. demonstrated that cavity-resident macrophages express high levels of TIM4, which sequesters highly cytotoxic PS-high CD8⁺ T cells and suppresses their proliferation, thereby inhibiting anti-tumor immune responses within the pleural and peritoneal tumor microenvironments [11]. Notably, in the present study, TIM4-positive macrophage infiltration was frequently observed in regions with dense lymphocyte accumulation. Taken together, these findings suggest that TIM4-positive macrophages may contribute to breast cancer progression by interacting with immune cells and suppressing tumor immunity. Another possible mechanism is that TIM4-positive macrophages may directly influence cancer cells through the secretion of soluble factors. TIM4 has been reported to promote epithelial-to-mesenchymal transition and exacerbate chronic rhinosinusitis with nasal polyps by enhancing the production of transforming growth factor-beta 1 (TGF-β1) in macrophages [38]. TGF-β1 is well known to induce tumor-promoting functions in macrophages and to directly stimulate the growth of breast cancer cells [39, 40]. Therefore, soluble factors such as TGF-β1 secreted by TIM4-positive macrophages may contribute to breast cancer cell proliferation and invasion. Although univariate analysis identified infiltration of TIM4-positive macrophages a as a prognostic factor, multivariate analysis showed that infiltration of TIM4-positive macrophages was not an independent prognostic factor, whereas pT remained an independent prognostic factor and Ki-67 also tended to be an independent prognostic factor for disease-free survival (P = 0.067). In the present study, infiltration of TIM4-positive macrophages was strongly correlated with Ki-67, and as mentioned above, soluble factors such as TGF-β1 secreted by TIM4-positive macrophages may contribute to breast cancer cell proliferation and invasion. Therefore, the results of the multivariate analysis showing that pT and Ki-67 tended to be independent prognostic predictors may partially reflect the role of TIM4-positive macrophages in promoting breast cancer cell proliferation and invasion.

In the present study, we did not find a significant correlation between TIM4 immunoreactivity in carcinoma cells and poor clinical outcomes of breast cancer patients, although previous reports have shown that TIM4 expression in carcinoma cells is associated with worse prognosis in lung cancer, PDAC, and CCRCC [13, 14, 29, 30]. Previous studies have reported that TIM4 expression in carcinoma cells is associated with larger tumor size, increased risk of lymph node metastasis, and poor histological differentiation, and promotes cancer progression and metastasis by regulating mitochondrial homeostasis in non-small cell lung cancer, based on immunohistochemistry and cell line investigations [13–16]. In contrast, no significant correlation was found between TIM4 expression and clinicopathological factors in CCRCC [29]. While the role of TIM4 in cancer cells appears to differ across cancer types, it has been suggested that TIM4 expression in breast cancer cells may not have a significant impact on tumor progression.

A limitation of this study is that we were unable to demonstrate the biological function of TIM4-positive macrophages. Although immunohistochemistry of clinical specimens suggested that TIM4-positive macrophages may act as a poor prognostic factor, this finding remains speculative, and further studies using cell lines and mouse models are required to clarify the causal relationship. In addition, while this study examined the colocalization of TIM4 with several immune cell markers using multiplex immunohistochemistry, more detailed cell lineage analyses using flow cytometry and single-cell approaches are needed for further investigation. In addition, the breast cancer specimens used in the present study were derived entirely from a single institution over a limited time period, and further bioinformatics analyses using large-scale public datasets may help elucidate additional functions of TIM4 and clarify its reliability as a biomarker. In conclusion, TIM4 is expressed in both macrophages and carcinoma cells in breast cancer tissues, and infiltration of TIM4-positive macrophages serves as a strong prognostic predictor for breast cancer. TIM4 may represent a novel marker for tumor-promoting macrophages, and further studies are needed to clarify its functional role.

## Supplementary Information

Below is the link to the electronic supplementary material.

Supplementary file1 (PPTX 238 KB)

Supplementary file2 (DOCX 39 KB)

## Data Availability

All data and materials in the article are available from the corresponding author upon reasonable request.

## References

[1] Bray F, Laversanne M, Sung H, Ferlay J, Siegel RL, Soerjomataram I, et al. Global cancer statistics 2022: GLOBOCAN estimates of incidence and mortality worldwide for 36 cancers in 185 countries. CA Cancer J Clin. 2024;74:229–63. 10.3322/caac.21834. 38572751 10.3322/caac.21834

[2] Zgura A, Galesa L, Bratila E, Anghel R. Relationship between tumor infiltrating lymphocytes and progression in breast cancer. Maedica (Bucur). 2018;13:317–20. 10.26574/maedica.2018.13.4.317. 30774731 10.26574/maedica.2018.13.4.317 PMC6362880

[3] Yamaguchi-Tanaka M, Takagi K, Sato A, Yamazaki Y, Miyashita M, Masamune A, et al. Regulation of stromal cells by sex steroid hormones in the breast cancer microenvironment. Cancers (Basel). 2024;16:4043. 10.3390/cancers16234043. 39682229 10.3390/cancers16234043 PMC11639972

[4] Miyanishi M, Tada K, Koike M, Uchiyama Y, Kitamura T, Nagata S. Identification of Tim4 as a phosphatidylserine receptor. Nature. 2007;450:435–9. 10.1038/nature06307. 17960135 10.1038/nature06307

[5] Wang Z, Chen C, Su Y, Ke N. Function and characteristics of TIM‑4 in immune regulation and disease (Review). Int J Mol Med. 2023;51:10. 10.3892/ijmm.2022.5213. 36524355 10.3892/ijmm.2022.5213 PMC9848438

[6] Meyers JH, Chakravarti S, Schlesinger D, Illes Z, Waldner H, Umetsu SE, et al. TIM-4 is the ligand for TIM-1, and the TIM-1–TIM-4 interaction regulates T cell proliferation. Nat Immunol. 2005;6:455–64. 10.1038/ni1185. 15793576 10.1038/ni1185

[7] Wang Y, Wang Y, Ge Y, Wu Z, Yue X, Li C, et al. Tim-4 alleviates acute hepatic injury by modulating homeostasis and function of NKT cells. Clin Exp Immunol. 2024;218:101–10. 10.1093/cei/uxae063. 39036980 10.1093/cei/uxae063 PMC11404119

[8] Ding Q, Mohib K, Kuchroo VK, Rothstein DM. TIM-4 identifies IFN-gamma-expressing proinflammatory B effector 1 cells that promote tumor and allograft rejection. J Immunol. 2017;199:2585–95. 10.4049/jimmunol.1602107. 28848066 10.4049/jimmunol.1602107 PMC5726915

[9] Baghdadi M, Yoneda A, Yamashina T, Nagao H, Komohara Y, Nagai S, et al. TIM-4 glycoprotein-mediated degradation of dying tumor cells by autophagy leads to reduced antigen presentation and increased immune tolerance. Immunity. 2013;39:1070–81. 10.1016/j.immuni.2013.09.014. 24315994 10.1016/j.immuni.2013.09.014

[10] Xia H, Li S, Li X, Wang W, Bian Y, Wei S, et al. Autophagic adaptation to oxidative stress alters peritoneal residential macrophage survival and ovarian cancer metastasis. JCI Insight. 2020;5:e141115. 10.1172/jci.insight.141115. 32780724 10.1172/jci.insight.141115 PMC7526547

[11] Chow A, Schad S, Green MD, Hellmann MD, Allaj V, Ceglia N, et al. Tim-4(+) cavity-resident macrophages impair anti-tumor CD8(+) T cell immunity. Cancer Cell. 2021;39:973-88.e9. 10.1016/j.ccell.2021.05.006. 34115989 10.1016/j.ccell.2021.05.006 PMC9115604

[12] Baghdadi M, Nagao H, Yoshiyama H, Akiba H, Yagita H, Dosaka-Akita H, et al. Combined blockade of TIM-3 and TIM-4 augments cancer vaccine efficacy against established melanomas. Cancer Immunol Immunother. 2013;62:629–37. 10.1007/s00262-012-1371-9. 23143694 10.1007/s00262-012-1371-9 PMC11029366

[13] Zhang Q, Wang H, Wu X, Liu B, Liu W, Wang R, et al. TIM-4 promotes the growth of non-small-cell lung cancer in a RGD motif-dependent manner. Br J Cancer. 2015;113:1484–92. 10.1038/bjc.2015.323. 26512878 10.1038/bjc.2015.323 PMC4815884

[14] Liu W, Wang H, Bai F, Ding L, Huang Y, Lu C, et al. IL-6 promotes metastasis of non-small-cell lung cancer by up-regulating TIM-4 via NF-kappaB. Cell Prolif. 2020;53:e12776. 10.1111/cpr.12776. 32020709 10.1111/cpr.12776 PMC7106962

[15] Chen S, Wang Y, Liu W, Liang Y, Wang Y, Wu Z, et al. N-glycosylation at Asn291 stabilizes TIM-4 and promotes the metastasis of NSCLC. Front Oncol. 2022;12:730530. 10.3389/fonc.2022.730530. 35433445 10.3389/fonc.2022.730530 PMC9008408

[16] Wang Y, Wang Y, Liu W, Ding L, Zhang X, Wang B, et al. TIM-4 orchestrates mitochondrial homeostasis to promote lung cancer progression via ANXA2/PI3K/AKT/OPA1 axis. Cell Death Dis. 2023;14:141. 10.1038/s41419-023-05678-3. 36806050 10.1038/s41419-023-05678-3 PMC9941510

[17] Khalid F, Takagi K, Sato A, Yamaguchi M, Guestini F, Miki Y, et al. Interleukin (IL)-17A in triple-negative breast cancer: a potent prognostic factor associated with intratumoral neutrophil infiltration. Breast Cancer. 2023;30:748–57. 10.1007/s12282-023-01467-0. 37178415 10.1007/s12282-023-01467-0

[18] Taguchi R, Yamaguchi-Tanaka M, Takagi K, Sato A, Miki Y, Miyashita M, et al. Clinicopathological significance and prognostic role of high mobility group box 1 (HMGB1), toll-like receptor (TLR) 2 and TLR4 in breast cancer. Acta Histochem Cytochem. 2024;57:75–83. 10.1267/ahc.24-00006. 38695037 10.1267/ahc.24-00006 PMC11058461

[19] Hamlin IM. Possible host resistance in carcinoma of the breast: a histological study. Br J Cancer. 1968;22:383–401. 10.1038/bjc.1968.47. 5681002 10.1038/bjc.1968.47 PMC2008376

[20] Yamaguchi M, Takagi K, Sato M, Sato A, Miki Y, Onodera Y, et al. Androgens enhance the ability of intratumoral macrophages to promote breast cancer progression. Oncol Rep. 2021;46:188. 10.3892/or.2021.8139. 34278480 10.3892/or.2021.8139

[21] Shin SJ, DeLellis RA, Ying L, Rosen PP. Small cell carcinoma of the breast: a clinicopathologic and immunohistochemical study of nine patients. Am J Surg Pathol. 2000;24:1231–8. 10.1097/00000478-200009000-00006. 10976697 10.1097/00000478-200009000-00006

[22] Zhao S, Ma D, Xiao Y, Li XM, Ma JL, Zhang H, et al. Molecular subtyping of triple-negative breast cancers by immunohistochemistry: molecular basis and clinical relevance. Oncologist. 2020;25:e1481–91. 10.1634/theoncologist.2019-0982. 32406563 10.1634/theoncologist.2019-0982 PMC7543239

[23] Popa O, Taban SM, Pantea S, Plopeanu AD, Barna RA, Cornianu M, et al. The new WHO classification of gastrointestinal neuroendocrine tumors and immunohistochemical expression of somatostatin receptor 2 and 5. Exp Ther Med. 2021;22:1179. 10.3892/etm.2021.10613. 34475969 10.3892/etm.2021.10613 PMC8406677

[24] Kobayashi N, Karisola P, Peña-Cruz V, Dorfman DM, Jinushi M, Umetsu SE, et al. TIM-1 and TIM-4 glycoproteins bind phosphatidylserine and mediate uptake of apoptotic cells. Immunity. 2007;27:927–40. 10.1016/j.immuni.2007.11.011. 18082433 10.1016/j.immuni.2007.11.011 PMC2757006

[25] Wong K, Valdez PA, Tan C, Yeh S, Hongo JA, Ouyang W. Phosphatidylserine receptor Tim-4 is essential for the maintenance of the homeostatic state of resident peritoneal macrophages. Proc Natl Acad Sci U S A. 2010;107:8712–7. 10.1073/pnas.0910929107. 20421466 10.1073/pnas.0910929107 PMC2889355

[26] Zhang X, Gu J, Zhou L, Mi QS. TIM-4 is expressed on invariant NKT cells but dispensable for their development and function. Oncotarget. 2016;7:71099–111. 10.18632/oncotarget.12153. 27662666 10.18632/oncotarget.12153 PMC5340118

[27] Li J, Cao D, Guo G, Wu Y, Chen Y. Expression and anatomical distribution of TIM-containing molecules in Langerhans cell sarcoma. J Mol Histol. 2013;44:213–20. 10.1007/s10735-012-9475-2. 23264111 10.1007/s10735-012-9475-2

[28] Li W, Li X, Xu S, Ma X, Zhang Q. Expression of Tim4 in glioma and its regulatory role in LN-18 glioma cells. Med Sci Monit. 2016;22:77–82. 10.12659/msm.894963. 26741116 10.12659/MSM.894963 PMC4710195

[29] Yano H, Motoshima T, Ma C, Pan C, Yamada S, Nakayama T, et al. The significance of TIMD4 expression in clear cell renal cell carcinoma. Med Mol Morphol. 2017;50:220–6. 10.1007/s00795-017-0164-9. 28631038 10.1007/s00795-017-0164-9

[30] Wang Z, Xie Z, Mou Y, Geng R, Chen C, Ke N. TIM-4 increases the proportion of CD4(+)CD25(+)FOXP3(+) regulatory T cells in the pancreatic ductal adenocarcinoma microenvironment by inhibiting IL-6 secretion. Cancer Med. 2024;13:e70110. 10.1002/cam4.70110. 39235042 10.1002/cam4.70110 PMC11375529

[31] Caronni N, Piperno GM, Simoncello F, Romano O, Vodret S, Yanagihashi Y, et al. TIM4 expression by dendritic cells mediates uptake of tumor-associated antigens and anti-tumor responses. Nat Commun. 2021;12:2237. 10.1038/s41467-021-22535-z. 33854047 10.1038/s41467-021-22535-z PMC8046802

[32] Murray PJ, Allen JE, Biswas SK, Fisher EA, Gilroy DW, Goerdt S, et al. Macrophage activation and polarization: nomenclature and experimental guidelines. Immunity. 2014;41:14–20. 10.1016/j.immuni.2014.06.008. 25035950 10.1016/j.immuni.2014.06.008 PMC4123412

[33] Thornley TB, Fang Z, Balasubramanian S, Larocca RA, Gong W, Gupta S, et al. Fragile TIM-4-expressing tissue resident macrophages are migratory and immunoregulatory. J Clin Invest. 2014;124:3443–54. 10.1172/JCI73527. 24983317 10.1172/JCI73527 PMC4109530

[34] Ding L, Liang Y, Wang Y, Tong Z, Liu W, Tan S, et al. T-cell immunoglobulin- and mucin-domain-containing molecule-4 maintains adipose tissue homeostasis by orchestrating M2 macrophage polarization via nuclear factor kappa B pathway. Immunology. 2023;168:49–62. 10.1111/imm.13555. 35908188 10.1111/imm.13555

[35] Liang X, Luo M, Shao B, Yang JY, Tong A, Wang RB, et al. Phosphatidylserine released from apoptotic cells in tumor induces M2-like macrophage polarization through the PSR-STAT3-JMJD3 axis. Cancer Commun (Lond). 2022;42:205–22. 10.1002/cac2.12272. 35191227 10.1002/cac2.12272 PMC8923121

[36] Elfstrum AK, Bapat AS, Schwertfeger KL. Defining and targeting macrophage heterogeneity in the mammary gland and breast cancer. Cancer Med. 2024;13:e7053. 10.1002/cam4.7053. 38426622 10.1002/cam4.7053 PMC10905685

[37] Kumar Jha P, Aikawa M, Aikawa E. Macrophage Heterogeneity and Efferocytosis: Beyond the M1/M2 Dichotomy. Circ Res. 2024;134:186–8. 10.1161/CIRCRESAHA.123.324011. 38236949 10.1161/CIRCRESAHA.123.324011 PMC10798221

[38] Qin D, Liu P, Zhou H, Jin J, Gong W, Liu K, et al. TIM-4 in macrophages contributes to nasal polyp formation through the TGF-beta1-mediated epithelial to mesenchymal transition in nasal epithelial cells. Front Immunol. 2022;13:941608. 10.3389/fimmu.2022.941608. 35990621 10.3389/fimmu.2022.941608 PMC9389014

[39] de Kruijf EM, Dekker TJA, Hawinkels LJAC, Putter H, Smit VTHBM, Kroep JR, et al. The prognostic role of TGF-beta signaling pathway in breast cancer patients. Ann Oncol 2013;24:384–90. 10.1093/annonc/mds333. 10.1093/annonc/mds333 23022998

[40] Yamaguchi-Tanaka M, Takagi K, Miki Y, Sato A, Iwabuchi E, Miyashita M, et al. The pro-tumorigenic role of chemotherapy-induced extracellular HSP70 from breast cancer cells via intratumoral macrophages. Cancers (Basel). 2023;15:1903. 10.3390/cancers15061903. 36980788 10.3390/cancers15061903 PMC10047178

