# Supplementary material for: Infiltration of TIM4-positive intratumoral macrophages serves as an adverse prognostic factor in breast cancer
Source: Breast Cancer. 2026 Jan 21;33(2):436–47. doi: 10.1007/s12282-026-01825-8 (PMC12960482; doi:10.1007/s12282-026-01825-8)
Supplement: Supplementary file 1 — Supplementary file1 (PPTX 238 KB) [file 12282_2026_1825_MOESM1_ESM.pptx]

## Slide 1
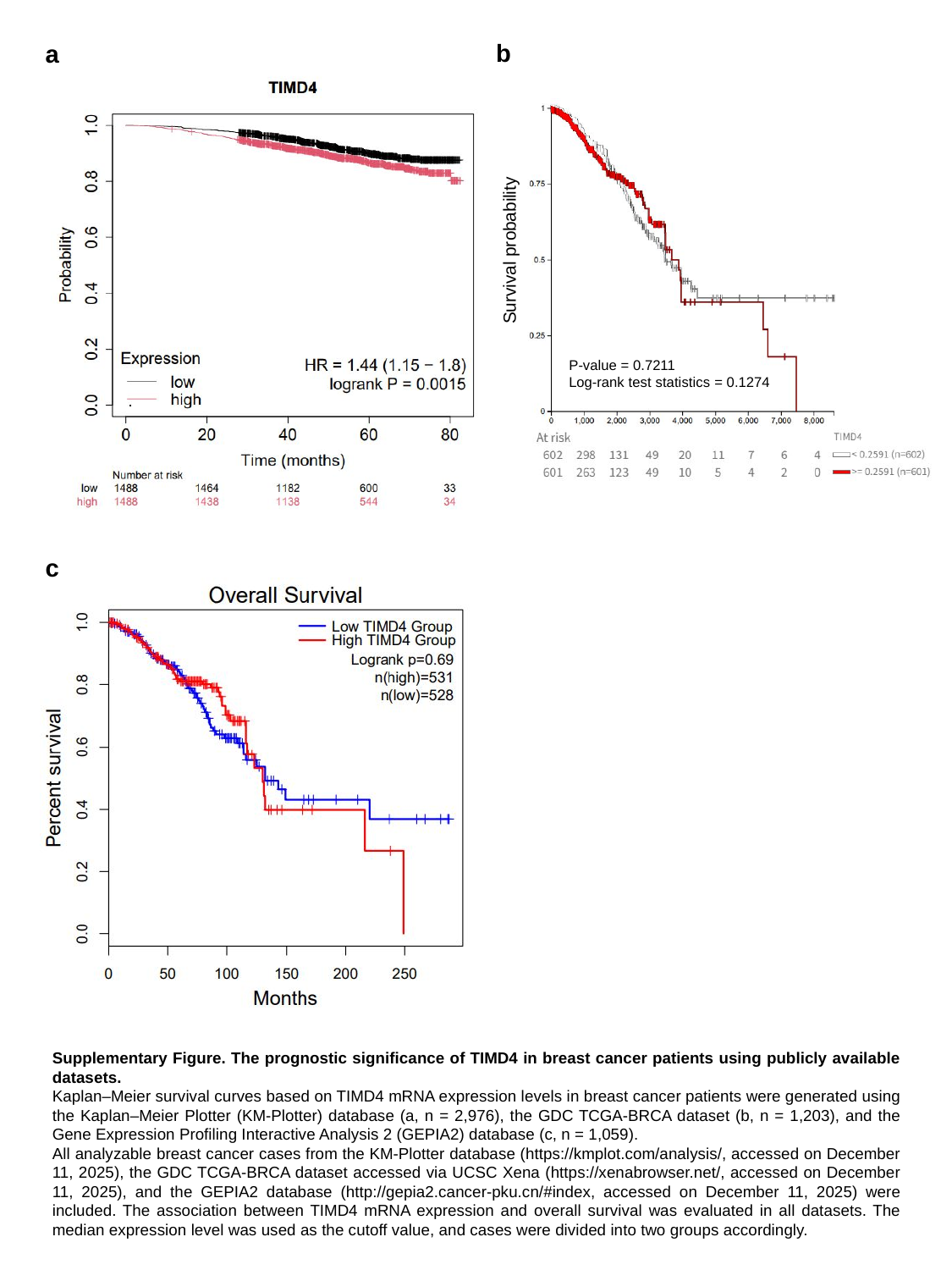

b
a
Survival probability
P-value = 0.7211
Log-rank test statistics = 0.1274
c
Supplementary Figure. The prognostic significance of TIMD4 in breast cancer patients using publicly available datasets.
Kaplan–Meier survival curves based on TIMD4 mRNA expression levels in breast cancer patients were generated using the Kaplan–Meier Plotter (KM-Plotter) database (a, n = 2,976), the GDC TCGA-BRCA dataset (b, n = 1,203), and the Gene Expression Profiling Interactive Analysis 2 (GEPIA2) database (c, n = 1,059).
All analyzable breast cancer cases from the KM-Plotter database (https://kmplot.com/analysis/, accessed on December 11, 2025), the GDC TCGA-BRCA dataset accessed via UCSC Xena (https://xenabrowser.net/, accessed on December 11, 2025), and the GEPIA2 database (http://gepia2.cancer-pku.cn/#index, accessed on December 11, 2025) were included. The association between TIMD4 mRNA expression and overall survival was evaluated in all datasets. The median expression level was used as the cutoff value, and cases were divided into two groups accordingly.
