# Supplementary material for: Infiltration of TIM4-positive intratumoral macrophages serves as an adverse prognostic factor in breast cancer
Source: Breast Cancer. 2026 Jan 21;33(2):436–47. doi: 10.1007/s12282-026-01825-8 (PMC12960482; doi:10.1007/s12282-026-01825-8)
Supplement: Supplementary file 2 — Supplementary file2 (DOCX 39 KB) [file 12282_2026_1825_MOESM2_ESM.docx]

**Supplementary Table. Uni- and multivariate analysis of disease-free and breast cancer-specific survival in 171 breast cancer patients.**

|  | **Disease-free survival** | | | | |  | **Breast cancer-specific survival** | | | | |
| --- | --- | --- | --- | --- | --- | --- | --- | --- | --- | --- | --- |
|  | **Univariate** | |  | **Multivariate** | |  | **Univariate** | |  | **Multivariate** | |
|  | P | relative risk  (95% CI) |  | P | relative risk  (95% CI) |  | P | relative risk  (95% CI) |  | P | relative risk  (95% CI) |
| pT  (pT2-4/pT1) | **< 0.001** | **5.6**  **(2.6–12)** |  | **0.0015** | **4.3**  **(1.7–11)** |  | **0.0001** | **11**  **(2.5–51)** |  | **0.018** | **6.2**  **(1.2–33)** |
| Lymph node metastasis  (Positive/Negative) | **< 0.0023** | **3.0**  **(1.5–6.2)** |  | 0.84 | 1.1  (0.47–2.5) |  | **0.0031** | **5.4**  **(1.7–18)** |  | 0.33 | 1.9  (0.50–7.3) |
| Histological grade  (3/1+2) | **0.021** | **2.5**  **(1.2–5.3)** |  | 0.20 | 0.51  (0.18–1.4) |  | **0.018** | **4.1**  **(1.3–13)** |  | 0.48 | 1.8  (0.36–8.5) |
| ER  (Negative/Positive) | **0.011** | **2.8**  **(1.3–5.8)** |  | 0.55 | 1.4  (0.47–4.1) |  | 0.079 | 2.9  (0.95–8.9) |  | - | - |
| PR  (Negative/Positive) | **0.0009** | **3.3**  **(1.6–6.9)** |  | 0.093 | 2.3  (0.90–5.8) |  | **0.010** | **4.3**  **(1.3–14)** |  | 0.085 | 3.4  (0.80–15) |
| HER2  (Negative/Positive) | 0.064 | 3.1  (0.75–13) |  | - | - |  | 0.30 | 2.6  (0.33–20) |  | - | - |
| Ki67 LI  (≥20%/<20%) | **< 0.0001** | **4.5**  **(2.2–9.3)** |  | 0.067 | 2.6  (0.96–7.1) |  | **0.0053** | **4.8**  **(1.6–15)** |  | 0.81 | 0.80  (0.12–5.1) |
| TIM4-stroma  (High/Low) | **0.015** | **2.9**  **(1.3–6.2)** |  | 0.36 | 1.5  (0.63–3.6) |  | **0.042** | **3.5**  **(1.1–11)** |  | 0.24 | 2.3  (0.59–8.7) |

Both univariate and multivariate analyses were performed using the Cox proportional hazards model. P < 0.05 was considered significant and examined in multivariate analysis and described as bold. HER2; human epidermal growth factor receptor type 2, LI; labeling index, pT; pathological T factor.
